# In Vitro and In Silico Acetylcholinesterase Inhibitory Activity of Thalictricavine and Canadine and Their Predicted Penetration across the Blood-Brain Barrier

**DOI:** 10.3390/molecules24071340

**Published:** 2019-04-05

**Authors:** Jakub Chlebek, Jan Korábečný, Rafael Doležal, Šárka Štěpánková, Daniel I. Pérez, Anna Hošťálková, Lubomír Opletal, Lucie Cahlíková, Kateřina Macáková, Tomáš Kučera, Martina Hrabinová, Daniel Jun

**Affiliations:** 1 ADINACO Research Group, Department of Pharmaceutical Botany, Faculty of Pharmacy, Charles University, Akademika Heyrovského 1203, 500 05 Hradec Králové, Czech Republic; HOSTA4AA@faf.cuni.cz (A.H.); opletal@faf.cuni.cz (L.O.); cahlikova@faf.cuni.cz (L.C.); macakovak@faf.cuni.cz (K.M.); 2 Biomedical Research Center, University Hospital Hradec Králové, Sokolská 581, 500 05 Hradec Králové, Czech Republic; korabecny.jan1@gmail.com (J.K.); rafael.dolezal@gmail.com (R.D.); martina.hrabinova@unob.cz (M.H.); daniel.jun@unob.cz (D.J.); 3 Department of Toxicology and Military Pharmacy, Faculty of Military Health Sciences, University of Defense, Třebešská 1575, 500 01 Hradec Králové, Czech Republic; tomas.kucera2@unob.cz; 4 Center for Basic and Applied Research, Faculty of Informatics and Management, University of Hradec Králové, Rokitanského 62, 50003 Hradec Králové, Czech Republic; 5 Department of Biological and Biochemical Sciences, Faculty of Chemical Technology, University of Pardubice, Studentská 95, 532 10 Pardubice, Czech Republic; Sarka.Stepankova@upce.cz; 6 Centro de Investigaciones Biológicas, Avenida Ramiro de Maetzu 9, 280 40 Madrid, Spain; dperez@cib.csic.es

**Keywords:** (+)-thalictricavine, (+)-canadine, cholinesterases, kinetic study, molecular docking, blood–brain barrier permeability

## Abstract

In recent studies, several alkaloids acting as cholinesterase inhibitors were isolated from *Corydalis cava* (Papaveraceae). Inhibitory activities of (+)-thalictricavine (**1**) and (+)-canadine (**2**) on human acetylcholinesterase (*h*AChE) and butyrylcholinesterase (*h*BChE) were evaluated with the Ellman’s spectrophotometric method. Molecular modeling was used to inspect the binding mode of compounds into the active site pocket of *h*AChE. The possible permeability of **1** and **2** through the blood–brain barrier (BBB) was predicted by the parallel artificial permeation assay (PAMPA) and logBB calculation. In vitro, **1** and **2** were found to be selective *h*AChE inhibitors with IC_50_ values of 0.38 ± 0.05 µM and 0.70 ± 0.07 µM, respectively, but against *h*BChE were considered inactive (IC_50_ values > 100 µM). Furthermore, both alkaloids demonstrated a competitive-type pattern of *h*AChE inhibition and bind, most probably, in the same AChE sub-site as its substrate. In silico docking experiments allowed us to confirm their binding poses into the active center of *h*AChE. Based on the PAMPA and logBB calculation, **2** is potentially centrally active, but for **1** BBB crossing is limited. In conclusion, **1** and **2** appear as potential lead compounds for the treatment of Alzheimer’s disease.

## 1. Introduction

Alzheimer’s disease (AD) is an age-related, progressive, neurodegenerative disorder, with onset in humans usually over the age of 65. The disease is characterized by cognitive impairment, a variety of behavioral symptoms, and restrictions in activities of daily living. The prevalence of AD increases exponentially between the ages of 65–85, doubling with every five-year age group [1]. Two characteristic pathological hallmarks can be found: extracellular deposition of β-amyloid peptide into amyloid plaques, and intraneuronal formation of hyperphosphorylated τ-protein filaments into neurofibrillary tangles, both causing progressive loss of neurons and disintegration of the neural circuits, particularly in the cerebral cortex [2,3]. A deficit in cholinergic functions and decreased levels of the neurotransmitter acetylcholine (ACh) in the cortex are responsible for the memory impairment in AD patients [4]. In this regard, acetylcholinesterase (AChE, E.C. 3.1.1.7), and also butyrylcholinesterase (BChE, E.C. 3.1.1.8) in later AD phases, hydrolyze ACh and terminate its action [5]. Therefore, to maintain ACh levels in synapses, cholinesterase inhibitors (ChEIs) are clinically used as AD therapeutics [6,7,8].

The portfolio of ChEIs is limited; the U.S. Food and Drug Administration has approved donepezil, galanthamine (Gal) and rivastigmine to treat the symptoms of mild to moderate AD [9]. From the pharmacological point of view, Gal and donepezil are considered as selective AChE inhibitors, while rivastigmine acts as a dual cholinesterase inhibitor [6,10,11]. Furthermore, huperzine A (Hup A), an AChE selective inhibitor, has been widely used in China for AD treatment [12]. AChEIs (inhibitors of acetylcholinesterase) are still being extensively investigated in the chase of new and more effective remedies against AD, and some are in different phases of clinical trials [7,8]. In addition to their cognitive effect, they are often endowed with other, often disease-modifying effects, coined mostly to neuroprotective abilities (e.g., influencing of amyloid precursor protein metabolism, neuroprotection mediated via agonizing nicotinic ACh receptors, modulation of muscarinic ACh receptors, and inhibition of *N*-methyl-d-aspartate receptors) [4,7,12,13]. Since some recently developed drugs based on anti-amyloid action failed in phase III clinical trials, ChEIs still represent a perspective venue for the development of novel entities for AD therapy [7].

Potent AChEIs have been found among natural products [14,15,16], with both Gal and Hup A being used in symptomatic AD therapy [9]. AChEIs of natural origin are still being extensively investigated to find more potent compounds with better pharmacotherapeutic characteristics. In recent studies, several alkaloids acting as ChEIs were isolated from *Corydalis cava* (L.) Schweigg. & Körte [17,18], which was used in Danish folk medicine for memory and cognition improvement [19].

One of the principal approaches in designing of novel and effective AChEIs with a broad biological profile is associated with computational methods in the so-called computer-aided drug design [20]. In silico methods contribute to enhancement of the understanding of AChE-ligand architecture of products of natural origin [21,22,23,24,25]. Molecular modeling techniques also aim to provide novel AChEIs with a better predicted pharmacokinetic profile and lower toxicity [26].

Finally, if compounds are to be considered as drug candidates for AD treatment, it is necessary to determine their ability to cross the blood–brain barrier (BBB), which is a crucial aspect of all potential substances for the treatment of CNS-related disorders. The BBB is a protective system composed of endothelial cells with tight junctions that prevent substances going from blood into the brain [27,28,29]. Molecules that are soluble in lipids are able to penetrate relatively easily through the BBB via lipid cell membranes. On the other hand, hydrophilic molecules cross the barrier by use of specialized carrier-mediated transport mechanisms only [30]. A parallel artificial membrane permeability assay (PAMPA) is a model assay for determination of penetration via the BBB. The PAMPA is a high throughput method developed for the prediction of passive permeability through biological membranes [31].

**Figure 1:**
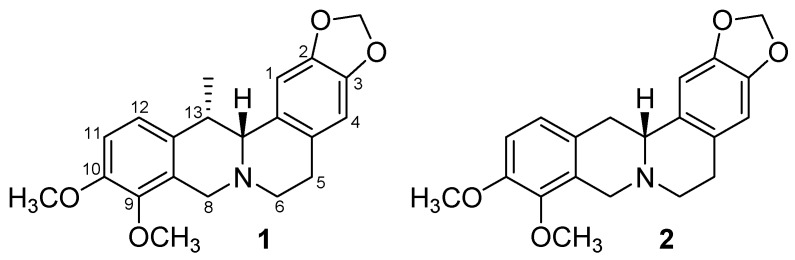
Structures of protoberberine alkaloids (+)-thalictricavine (**1**) and (+)-canadine (**2**).

This study is focused on the elucidation of the inhibition of human cholinesterases (*h*AChE and *h*BChE) by the protoberberine alkaloids (+)-thalictricavine (**1**) and (+)-canadine (**2**), previously isolated from *C. cava* (Figure 1) [17,32], and determination of their *h*AChE inhibition mechanism. For further insight into the experimental results, exploration of the binding mode into the active site pocket of human acetylcholinesterase (*h*AChE) was subsequently inspected by molecular modeling. The obtained data from the in silico assay were compared with Gal, the selective AChE inhibitor used in current AD therapy. Furthermore, the ability of alkaloids **1** and **2** to cross the BBB was predicted by the PAMPA to consider these compounds either as potential drugs or as lead scaffolds for the development of new potential substances for AD treatment.

## 2. Results and Discussion

### 2.1. AChE and BChE Inhibition Studies

Compounds **1** and **2**, which are found mainly in *Corydalis* species [17,32,33,34,35], and structurally almost identical (**1** possesses one methyl group extra at position 13 of the protoberberine skeleton in comparison with **2**; Figure 1), were tested for their ability to inhibit *h*AChE and *h*BChE; their IC_50_ values were determined, using Ellman’s method [36]. Recombinant *h*AChE and plasma *h*BChE were used as sources of cholinesterases, along with Gal as a positive standard. Furthermore, the AChE selectivity index (SI) was calculated for both compounds (Table 1). 

Both compounds demonstrated significant and more selective inhibition of *h*AChE in comparison with Gal, but against *h*BChE were considered inactive with IC_50_ values > 100 µM. Whereas the cholinesterase inhibition activity of **1** is reported in this study for the first time, **2** was screened for this activity previously and demonstrated an IC_50_ value for AChE in the low micromolar range [17]. The contrasting results for AChE inhibition of **2** could be explained by using different conditions in the assay such as concentration of the substrate, acetylthiocholine (ATCh), room temperature (25 °C), and a source of AChE (red blood cell ghosts) with unknown enzyme activity. Interestingly, the (+)-form of these tetrahydroprotoberberines is responsible for their significant *h*AChE inhibition: the (−)-form of **2** is considered inactive (*h*AChE IC_50_ = 637 ± 83 µM) [37]. Furthermore, the presence of the methyl group at position 13 in the structure of **1** slightly increases the potency of AChE inhibition as compared with **2** (Table 1). As mentioned above, AChE and BChE play important roles in the pathophysiology of AD, and thus cholinesterase inhibitors are used in current therapy. However, the cholinesterase inhibitors proposed for treating dementia in AD should demonstrate selective *h*AChE inhibition to minimalize their unwanted cholinergic peripheral side effects, which are associated with BChE inhibition [38]. Additionally, to consider compounds potential anti-Alzheimer’s disease drugs, candidates do not have to possess any significant toxic effect. In a recent study by Chlebek et al., **1** and **2** were determined as non-cytototoxic (IC_50_ values > 10 µM) against selected human carcinoma and non-carcinoma cells [39]. Furthermore, in vivo, it was found that tetrahydroprotoberberine alkaloids (e.g., (±)-form and (−)-form of **2** possess low acute toxicity, their LD_50_ values in mice by oral administration were 940 and > 2000 mg/kg, respectively [40,41]. Thus, potent and selective AChEIs with low toxicity such as **1** and **2** may appear as potential lead compounds for the treatment of Alzheimer’s disease.

### 2.2. AChE Kinetic Studies

**Figure 2:**
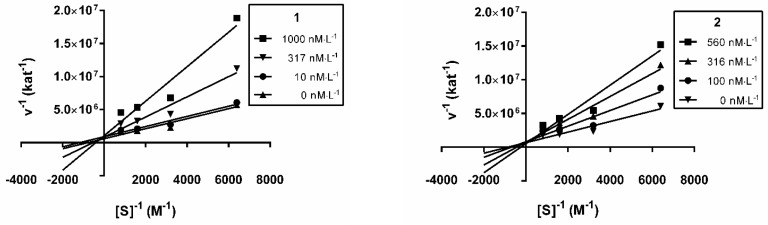
Steady-state inhibition of *h*AChE hydrolysis of acetylthiocholine by **1** and **2**. Lineweaver-Burk reciprocal plots of initial velocity and different substrate concentrations (156–1250 µM) are presented. Lines were derived from a weighted least-squares analysis of data.

The mode of interaction of the *h*AChE inhibition was elucidated for **1** and **2** using a kinetic study in order to gain information about the mode of inhibition and the binding site of the isolated alkaloids. Recombinant *h*AChE was used in this kinetic analysis of **1** and **2**. The mechanism of the inhibition process was examined by recording the set of substrate concentration—enzyme velocity curves in the presence of various concentrations of compounds **1** and **2**. The type of the inhibition mechanism was clarified by a nonlinear regression analysis. The results computed for each model type of inhibition (competitive, non-competitive, uncompetitive and mixed) were compared with the sum-of-squares F-test. The analysis confirmed a competitive type of inhibition (*p* ˂ 0.05) for both compounds, consistent with the graphical representation in Figure 2, showing Lineweaver−Burk reciprocal plots of the measured data [42]. Therefore, both alkaloids bind, most probably, in the same site as the *h*AChE substrate. With increasing concentration of inhibitors, the apparent V_max_ remained unchanged and K_m_ increased. A K_i_ value of 174.3 ± 56.9 nM was estimated by the nonlinear regression analysis for **1** and 128.9 ± 44.0 nM for **2**.

### 2.3. Docking Studies

**Figure 3:**
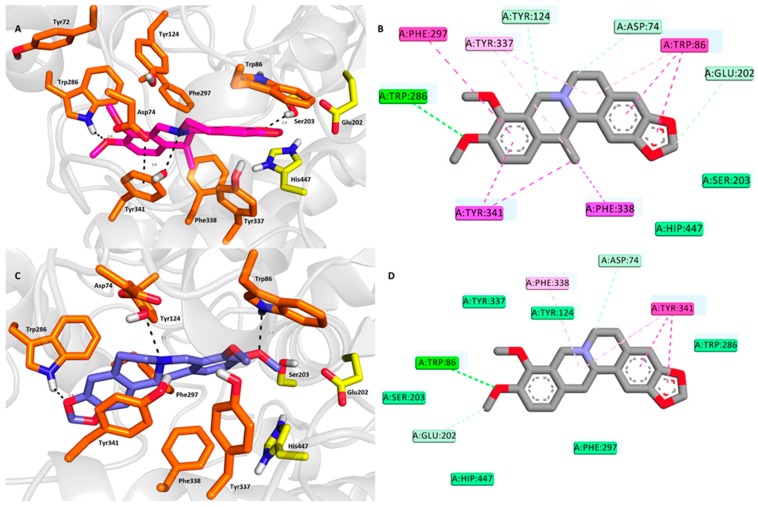
Top-scored docking poses for docking results for **1** and **2** within *h*AChE active site (PDB ID: 4EY6). (**A**,**C**)—superimposed analogue **1** and **2** in purple and blue, respectively, as three-dimensional (3D) figures; (**B**,**D**)—two-dimensional (2D) representation of **1** and **2**, respectively. Generally, in (**A**,**C**)—important amino acid residues involved in the ligand-enzyme interactions are displayed as orange carbon atoms; catalytic triad residues (Glu202, Ser203, His447) are shown in yellow, and the rest of the enzyme is represented as light-grey cartoon. (**B**,**D**) were created with Discovery Studio 2016 Client software; (**A**,**C**) were generated with PyMol 1.5.0.4 (The PyMOL Molecular Graphics System, Version 1.5.0.4 Schrödinger, LLC, Manheim, Germany).

The spatial conformation of **1** and **2** was explored by molecular modeling in order to reveal the differences in their binding modes to the *h*AChE active site (Protein Data Bank (PDB) ID: 4EY6). **1** adopted a binding pose in the proximity of the catalytic anionic site (CAS) of *h*AChE near the catalytic triad residues (Figure 3A,B). Accordingly, the hydroxyl residue of Ser203 is engaged in the hydrogen bond with the 1,3-dioxolan moiety of **1** (2.6 Å). In addition, Glu202, as well as His447, are also implicated in ligand anchoring via hydrophobic van der Waals forces. The protonated nitrogen of the 1,2,3,4-tetrahydroisoquinoline heterocycle of **1** provided another hydrogen contact to the hydroxyl of Tyr341 (3.5 Å). Moreover, Tyr341 (3.8 Å), as well as Phe297 (4.4 Å) face the aromatic region of the 1,2,3,4-tetrahydroisoquinoline moiety in parallel and T-shaped π-π interactions, respectively. Several other hydrophobic interactions can be observed in the *h*AChE-**1** complex including π-alkyl contacts with Tyr337, Tyr341, and Trp86. Specific π-sigma interactions also contribute to ligand anchoring, as established between Tyr341 and Phe338 and the methyl appendage of **1**.

On the contrary, a 180° rotated accommodation of **2** in the *h*AChE active site was observed (Figure 3C,D). This is presumably the major factor responsible for the negligible difference in the affinity towards the enzyme. Trp86 (2.6 Å) formed hydrogen contact with oxygen from one of the methoxy groups. Similarly, Trp286 (2.4 Å), a key peripheral anionic site (PAS) residue, established a hydrogen bond with the oxygen from the 1,3-dioxolan moiety. Tyr341 (3.8 Å) is engaged in parallel π-π stacking with the 2*H*-1,3-benzodioxole moiety. All the catalytic triad residues are also involved in ligand anchoring, mostly via either van der Waals forces (Ser203 and His447) or a carbon-hydrogen bond (Glu202).

In both, the docking simulation successfully correlated well with the crystallographic data, as found for the Gal-*h*AChE complex [43]. However, some subtle discrepancies can be observed like hydrogen bond formation between the tertiary amine of Gal and Tyr337. As described above for **1** and **2** complexed with *h*AChE, Tyr337 is implicated only in hydrophobic interactions. Similarly, Glu202 anchored the Gal hydroxyl group via a hydrogen bond, whereas this catalytic triad residue enabled hydrophobic contact in the case of **1** and **2**. Additionally, the limitation of the computational methodology using Autodock Vina makes it more difficult to anticipate the distribution of structural waters in *h*AChE complexed with either **1** or **2** [44]. It is well known that structural waters specifically contribute to Gal anchoring in the *h*AChE active site. Given the subtle difference in the in vitro results for **1** and **2**, the structure similarities, together with the close calculated binding affinities (−12.5 kcal/mol and −12.2 kcal/mol for **1** and **2**, respectively) it cannot be established which of these two ligands better fits into the enzyme active site. Last, but not least, both ligand spatial orientations provided an interesting platform for synthetic modifications of these two ligands in order to escalate their inhibition potency.

### 2.4. BBB Permeability

The ability of compounds to cross the BBB to reach their therapeutic targets in the CNS is the critical step in the development of new potential AD drugs. Thus, the screening for BBB penetration in the early drug discovery process provides important information for compound selection [45]. In order to explore the capacity of **1** and **2** to penetrate into the brain, the PAMPA-BBB method was used, which employed a brain lipid porcine membrane [31]. The in vitro permeability (*Pe*) of **1** and **2** through the lipid membrane extract was determined, together with nine commercial drugs used in an experiment validation (Supplementary material, Table S1). The validation was performed previously by comparison of the reported permeability values of commercial drugs with the experimental data obtained employing this methodology [46]. Based on the obtained results from the validation and following the pattern established in the literature for BBB permeation prediction [45], compound **2** could be classified as centrally active, whereas BBB crossing for **1** is limited (Table 1). Obviously, the PAMPA assay uses artificial membranes to observe passive membrane permeability, neglecting the special characteristics of the BBB. Therefore, we also decided to exploit another methodology calculating logBB for the compounds **1** and **2**. LogBB is the most common numeric value describing permeability across the BBB. It is defined as the logarithmic ratio between the concentration of a compound in the brain and blood [47]. The calculated logBB of **1** and **2** were −0.100 and 0.018, respectively (Table 1). Compounds with logBB > 0.3 readily cross the BBB, while those with logBB < −1.0 are only poorly distributed to the brain [48]. Carpenter et al. found similar data for BBB prediction of compounds using logBB calculation: compounds with logBB > 0 cross the BBB, while compounds possessing negative logBB were considered as being unable to cross the BBB [49]. Thus, analogous results from the PAMPA assay and logBB calculation for prediction of BBB crossing by passive permeation were obtained for **1** and **2**.

## 3. Materials and Methods

### 3.1. Instruments

The spectrophotometric readings for the *h*AChE and *h*BChE assays, and the kinetic study of *h*AChE inhibition were performed on a Multi-mode microplate reader Synergy^TM^ 2 (BioTek, Winooski, VT, USA), while a 96-well plate UV reader (Thermo Scientific, Multiskan spectrum, Vantaa, Finland) was used for the UV measurements of the PAMPA assay.

### 3.2. Materials and Chemicals

Human recombinant acetylcholinesterase (*h*AChE; EC 3.1.1.7), human plasma butyrylcholinesterase (*h*BChE; EC 3.1.1.8), 5,5′-dithiobis(2-nitrobenzoic acid) (Ellman’s reagent, DTNB), phosphate buffer (PB, pH 7.4), dimethyl sulfoxide (DMSO), acetylthiocholine (ATCh), and butyrylthiocholine (BTCh) were purchased from Sigma-Aldrich (Prague, Czech Republic). Polystyrene Nunc 96-well microplates with flat bottom shape (ThermoFisher Scientific, Waltham, MA, USA) were utilized for measuring purposes of cholinesterase assays and the kinetic inhibition study. (+)-Thalictricavine (**1**) and (+)-canadine (**2**) (≥98% by ^1^H-NMR spectroscopy) were previously isolated from *C. cava* [17,32]. Galanthamine hydrobromide 95% was purchased from Changsha Organic Herb Inc. (Changsha, China).

Nine commercially available drugs (caffeine, enoxacine, hydrocortisone, desipramine, piroxicam, testosterone, promazine, verapamil, and atenolol), phosphate buffer saline solution at pH 7.4 (PBS), ethanol, and dodecane were purchased from Sigma, Acros Organics, Merck), Aldrich, or Fluka (Madrid, Spain). The porcine polar brain lipid was from Avanti Polar Lipids (Madrid, Spain). The donor plate was a 96-well filtrate plate (Multiscreen IP sterile plate PDVF (polyvinylidene fluoride) membrane, pore size 0.45 μM) and the acceptor plate an indented 96-well plate (Multiscreen), purchased from Millipore. Filter PVDF membrane units (diameter 30 mm, pore size 0.45 μm) were purchased from Symta (Madrid, Spain).

### 3.3. AChE and BChE Inhibition

The *h*AChE and *h*BChE inhibitory activity of the tested compounds was determined using a modified Ellman’s method [36]. All the assays were carried out in 0.1 M KH_2_PO_4_/K_2_HPO_4_ buffer, pH 7.4. Enzyme solutions were prepared at an activity of 2.0 units/mL in 2 mL aliquots. The assay medium (100 µL) consisted of 40 µL of 0.1 M phosphate buffer (pH 7.4), 20 µL of 0.01 M DTNB, 10 µL of enzyme, and 20 µL of 0.01 M substrate (ATCh iodide solution). Assay solutions with the inhibitor (10 µL, 10^−3^–10^−9^ M) were preincubated for 5 min (the inhibitors were dissolved in DMSO and diluted in advance in the phosphate assay buffer to the desired concentration; the final concentration of DMSO did not exceed 0.1% in the assay medium). The reaction started by addition of 20 µL of the substrate (ATCh for *h*AChE, BTCh for *h*BChE). The enzyme activity was determined by measuring the increase in absorbance at 412 nm at 37 °C at 2 min intervals. Each concentration was assayed in triplicate. The obtained data were used to compute percentage of inhibition (*I*):

(1) $$I=\left(1-\frac{\Delta A_{i}}{\Delta A_{0}}\right)\times 100\;\;\;\left[\%\right]$$

where Δ*A_i_* indicates an absorbance change provided by the cholinesterase exposed to the cholinesterase inhibitor and Δ*A*_0_ indicates an absorbance change caused by the intact enzyme (the phosphate buffer was used instead of the inhibitor solution). Inhibition potency of tested compounds was expressed as an IC_50_ value (concentration of an inhibitor that causes 50% cholinesterase inhibition). Calculations were performed using software Microsoft Excel (Redmont, WA, USA) and GraphPad Prism version 6.07 for Windows, GraphPad Software, San Diego, CA, USA (www.graphpad.com).

### 3.4. Kinetic Study of AChE Inhibition

The kinetic study of *h*AChE inhibition was performed by using Ellman’s method (described above) [36]. For the measurements, the following concentrations of the substrate were used: 156, 313, 626 and 1250 µM. Reactions were performed in triplicate. V_max_ and K_m_ values, respectively, of the Michaelis-Menten kinetics and K_i_ were calculated by non-linear regression from the substrate velocity curves. Linear regression was used for calculation of Lineweaver-Burk plots. All calculations were performed using the GraphPad Prism software.

### 3.5. Molecular Docking Studies

From the online Protein Data Bank (PDB) database (www.pdb.org) the model of *h*AChE (PDB ID: 4EY6, resolution: 2.40 Å) was downloaded and prepared for flexible molecular docking by Molecular Graphics Laboratory (MGL) Tools utilities [43]. The preparation of this receptor involved removal of the surplus copies of the enzyme chains, non-bonded inhibitors, addition of polar hydrogens and merging of non-polar ones. Default Gasteiger charges were assigned to all atoms. The flexible parts of the enzymes were determined by a spherical selection of residues (R = 11 Å) approximately around the center of the active site. In the same points the center of the grid box of 33 × 33 × 33 Å was positioned. The rotatable bonds in the flexible residues were detected automatically by AutoDock Tools 1.5.4 program. Given the limitation of the program used for flexible molecular docking, water molecules had to be removed from the system. The flexible receptor parts contained 40 residues for *h*AChE. The following xyz coordinates of the grid box centers were applied: *h*AChE (10.698, −58.115, −23.192). The studied ligands were firstly drawn in HyperChem 8.0, then manually protonated as suggested by MarvinSketch 6.2.0. software (http://www.chemaxon.com), geometrically optimized by the semi-empirical quantum-chemistry PM3 method and stored as pdb files. The structures of the ligands were processed for docking in a similar way as the above mentioned flexible parts of the receptor by AutoDock Tools 1.5.4 program. Molecular docking was carried out in the AutoDock Vina 1.1.2 program utilizing the computer resources of the Czech National Grid Infrastructure MetaCentrum. The search algorithm of AutoDock Vina efficiently combines a Markov chain Monte Carlo like method for the global search and a Broyden-Fletcher-Goldfarb-Shano gradient approach for the local search [50]. It is a type of memetic algorithm based on interleaving stochastic and deterministic calculations [51]. Each docking task was repeated 15 times with the exhaustiveness parameter set to 16, employing 16 CPU in parallel multithreading. From the obtained results, the solutions reaching the minimum predicted Gibbs binding energy were taken as the top-scoring modes. The graphic representations of the docked poses were rendered in PyMol 1.5.0.4 (The PyMOL Molecular Graphics System, Version 1.5.0.4 Schrödinger, LLC, Mannheim, Germany). Two-dimensional (2D) diagrams were generated using Discovery Studio Visualizer v16.1.0.15350 (Dassault Systèmes Biovia Corp., 2016, San Diego, CA, USA).

### 3.6. Prediction of CNS Permeability

Prediction of brain penetration was evaluated using the PAMPA assay [31]. Test compounds (2–6 mg of caffeine, enoxacine, hydrocortisone, desipramine, piroxicam, testosterone; 12–15 mg of promazine and verapamil; and 23 mg of atenolol) were dissolved in EtOH (1000 μL). A 100 μL amount of each compound stock solution was taken and 1400 μL of EtOH and 3500 μL of PBS pH = 7.4 buffer were added to reach 30% EtOH concentration in the experiment. These solutions were filtered. The acceptor 96-well microplate was filled with 180 μL of PBS/EtOH (70:30). The donor 96-well plate was coated with 4 μL of porcine brain lipid in dodecane (20 mg mL^−1^), and, after 5 min, 180 μL of each compound solution was added. 1 to 2 mg of each test compound to be assayed for its ability to pass the brain barrier was dissolved in 1500 μL of EtOH and 3500 μL of PBS (pH 7.4) buffer, filtered, and then added to the donor 96-well plate. Then, the donor plate was carefully put on the acceptor plate to form a “sandwich”, which was left undisturbed for 2 h 45 min at 25 °C. During this time, the compounds diffused from the donor plate through the brain lipid membrane into the acceptor plate. After incubation, the donor plate was removed. The UV plate reader determined the concentration of compounds and clinically used drugs in the acceptor and donor wells.

## 4. Conclusions

Compounds **1** and **2** have promising and selective *h*AChE inhibition, which acts via a competitive inhibition mechanism. Docking experiments allowed us to estimate their binding poses into the active center of *h*AChE. Based on the PAMPA-BBB assay and logBB calculation, **2** is able to cross the BBB by passive permeation; however, for **1,** BBB crossing is limited. Considering all of the aforementioned, compounds **1** and **2** appear as either potential drugs or lead structures in the development of potent drugs for the treatment of AD.

## Figures and Tables

**Table 1:** *h*AChE and *h*BChE IC_50_ values of compounds for **1** and **2**, along with their *h*AChE selectivity and predicted permeabilities by the PAMPA-BBB assay and logBB calculation.

Compounds	IC_50_ ± SEM (µM) ^1^			PAMPA-BBB Permeability (*Pe*; 10^−6^ cm·s^−1^) ^3^	LogBB ^4^
	*h*AChE	*h*BChE	SI ^2^		
**1**	0.38 ± 0.05	>100	>263	2.5 ± 0.1 (CNS±)	−0.100
**2**	0.70 ± 0.07	>100	>143	5.0 ± 0.3 (CNS+)	0.018
Gal ^5^	0.26 ± 0.01	18.0 ± 1.90	69	n.d. ^6^	n.d. ^6^

^1^ Compound concentration required to decrease enzyme activity by 50%; IC_50_ values are the mean ± standard error of mean (SEM) of three independent measurements, each performed in triplicate. ^2^ Selectivity index for *h*AChE is determined as the ratio IC_50_ *_h_*_BChE_ / IC_50_ *_h_*_AChE_ values. ^3^ CNS (+): high BBB permeation predicted with *Pe* (10^−6^ cm·s^−1^) > 3.88, CNS (−): low BBB permeation predicted with *Pe* (10^−6^ cm·s^−1^) < 1.8, CNS (+/−): BBB permeation uncertain with *Pe* (10^−6^ cm·s^−1^) since 3.88 to 1.8. ^4^ calculated at http://www.way2drug.com/geb/. ^5^ Reference compound. ^6^ Not determined.

## Supplementary Materials

The following are available online, Table S1: Permeability in the PAMPA-BBB assay for nine commercial drugs (used in the experiment validation), compounds **1** and **2** and their predictive penetration into the CNS.

## References

[1] Lleó A. (2007). Current Therapeutic Options for Alzheimer’s Disease. Curr. Genom..

[2] Park S.-Y. (2010). Potential therapeutic agents against Alzheimer’s disease from natural sources. Arch. Pharm. Res..

[3] Rasool M., Malik A., Qureshi M.S., Manan A., Pushparaj P.N., Asif M., Qazi M.H., Qazi A.M., Kamal M.A., Gan S.H., Sheikh I.A. (2014). Recent updates in the treatment of neurodegenerative disorders using natural compounds. Evid. Based Complement. Alternat. Med..

[4] Lahiri D.K., Farlow M.R., Greig N.H., Sambamurti K. (2002). Current drug targets for Alzheimer’s disease treatment. Drug Dev. Res..

[5] Nordberg A., Ballard C., Bullock R., Darreh-Shori T., Somogyi M. (2013). A Review of butyrylcholinesterase as a therapeutic target in the treatment of Alzheimer’s Disease. Prim. Care Companion CNS Disord..

[6] Ehret M.J., Chamberlin K.W. (2015). Current practices in the treatment of Alzheimer disease: Where is the evidence after the Phase III trials?. Clin. Ther..

[7] Anand R., Gill K.D., Mahdi A.A. (2014). Therapeutics of Alzheimer’s disease: Past, present and future. Neuropharmacology.

[8] Schneider L.S., Mangialasche F., Andreasen N., Feldman H., Giacobini E., Jones R., Mantua V., Mecocci P., Pani L., Winblad B. (2014). Clinical trials and late-stage drug development for Alzheimer’s disease: an appraisal from 1984 to 2014. J. Intern. Med..

[9] Zemek F., Drtinova L., Nepovimova E., Sepsova V., Korabecny J., Klimes J., Kuca K. (2014). Outcomes of Alzheimer’s disease therapy with acetylcholinesterase inhibitors and memantine. Expert Opin. Drug Saf..

[10] Giacobini E. (2004). Cholinesterase inhibitors: new roles and therapeutic alternatives. Pharmacol. Res..

[11] Kandiah N., Pai M.-C., Senanarong V., Looi I., Ampil E., Park K.W., Karanam A.K., Christopher S. (2017). Rivastigmine: the advantages of dual inhibition of acetylcholinesterase and butyrylcholinesterase and its role in subcortical vascular dementia and Parkinson’s disease dementia. Clin. Interv. Aging.

[12] Zhang H. (2012). New insights into huperzine A for the treatment of Alzheimer’s disease. Acta Pharmacol. Sin..

[13] Zhang J.-M., Hu G.-Y. (2001). Huperzine A, a nootropic alkaloid, inhibits N-methyl-D-aspartate-induced current in rat dissociated hippocampal neurons. Neuroscience.

[14] Hostettmann K., Borloz A., Urbain A., Marston A. (2006). Natural product inhibitors of acetylcholinesterase. Curr. Org. Chem..

[15] Mukherjee P.K., Kumar V., Mal M., Houghton P.J. (2007). Acetylcholinesterase inhibitors from plants. Phytomedicine.

[16] Houghton P.J., Ren Y., Howes M.-J. (2006). Acetylcholinesterase inhibitors from plants and fungi. Nat. Prod. Rep..

[17] Chlebek J., Macáková K., Cahlíkovi L., Kurfürst M., Kunes J., Opletal L. (2011). Acetylcholinesterase and butyrylcholinesterase inhibitory compounds from *Corydalis cava* (Fumariaceae). Nat. Prod. Commun..

[18] Adsersen A., Kjølbye A., Dall O., Jäger A.K. (2007). Acetylcholinesterase and butyrylcholinesterase inhibitory compounds from *Corydalis cava* Schweigg. & Kort. J. Ethnopharmacol..

[19] Adsersen A., Gauguin B., Gudiksen L., Jäger A.K. (2006). Screening of plants used in Danish folk medicine to treat memory dysfunction for acetylcholinesterase inhibitory activity. J. Ethnopharmacol..

[20] Zeng H., Wu X. (2016). Alzheimer’s disease drug development based on computer-aided drug design. Eur. J. Med. Chem..

[21] Bermudez-Lugo J.A., Rosales-Hernandez M.C., Deeb O., Trujillo-Ferrara J., Correa-Basurto J. (2011). In silico methods to assist drug developers in acetylcholinesterase inhibitor design. Curr. Med. Chem..

[22] Ortiz J.E., Pigni N.B., Andujar S.A., Roitman G., Suvire F.D., Enriz R.D., Tapia A., Bastida J., Feresin G.E. (2016). Alkaloids from *Hippeastrum argentinum* and their cholinesterase-inhibitory activities: An in vitro and in silico study. J. Nat. Prod..

[23] Castillo-Ordóñez W.O., Tamarozzi E.R., da Silva G.M., Aristizabal-Pachón A.F., Sakamoto-Hojo E.T., Takahashi C.S., Giuliatti S. (2017). Exploration of the acetylcholinesterase inhibitory activity of some alkaloids from Amaryllidaceae family by molecular docking in silico. Neurochem. Res..

[24] Cortes N., Alvarez R., Osorio E.H., Alzate F., Berkov S., Osorio E. (2015). Alkaloid metabolite profiles by GC/MS and acetylcholinesterase inhibitory activities with binding-mode predictions of five Amaryllidaceae plants. J. Pharm. Biomed. Anal..

[25] Adhami H.R., Linder T., Kaehlig H., Schuster D., Zehl M., Krenn L. (2012). Catechol alkenyls from *Semecarpus anacardium*: Acetylcholinesterase inhibition and binding mode predictions. J. Ethnopharmacol..

[26] da Silva V.B., de Andrade P., Kawano D.F., Morais P.A.B., de Almeida J.R., Carvalho I., Taft C.A., da Silva C.H.T.D.P. (2011). In silico design and search for acetylcholinesterase inhibitors in Alzheimer’s disease with a suitable pharmacokinetic profile and low toxicity. Future Med. Chem..

[27] Pardridge W.M. (2001). Crossing the blood-brain barrier: are we getting it right?. Drug Discov. Today.

[28] Abbott N.J. (2013). Blood-brain barrier structure and function and the challenges for CNS drug delivery. J. Inherit. Metab. Dis..

[29] van Asperen J., Mayer U., van Tellingen O., Beijnen J.H. (1997). The functional role of P-glycoprotein in the blood-brain barrier. J. Pharm. Sci..

[30] Nielsen P.A., Andersson O., Hansen S.H., Simonsen K.B., Andersson G. (2011). Models for predicting blood-brain barrier permeation. Drug Discov. Today.

[31] Di L., Kerns E.H., Fan K., McConnell O.J., Carter G.T. (2003). High throughput artificial membrane permeability assay for blood-brain barrier. Eur. J. Med. Chem..

[32] Cahlíková L., Hulová L., Hrabinová M., Chlebek J., Hošťálková A., Adamcová M., Šafratová M., Jun D., Opletal L., Ločárek M. (2015). Isoquinoline alkaloids as prolyl oligopeptidase inhibitors. Fitoterapia.

[33] Slavík J., Slavíková L. (1979). Alkaloids from *Corydalis cava* (L.) SCHW. et KOERTE. Collect. Czech. Chem. Commun..

[34] Guo Z., Cai R., Su H., Li Y. (2014). Alkaloids in processed rhizoma *Corydalis* and crude rhizoma *Corydalis* analyzed by GC/MS. J. Anal. Methods Chem..

[35] guang Ma W., Fukushi Y., Tahara S. (1999). Fungitoxic alkaloids from Hokkaido *Corydalis* species. Fitoterapia.

[36] Ellman G.L., Courtney K.D., Andres V., Featherstone R.M. (1961). A new and rapid colorimetric determination of acetylcholinesterase activity. Biochem. Pharmacol..

[37] Hošťálková A., Kuneš J., Macáková K., Hrabinová M., Opletal L. (2015). Alkaloids from *Hydrastidis canadensis* and their cholinesterase and prolyl oligopeptidase inhibitory. Ceska Slov. Farm..

[38] Francis P.T., Palmer A.M., Snape M., Wilcock G.K. (1999). The cholinergic hypothesis of Alzheimer’s disease: a review of progress. J. Neurol. Neurosurg. Psychiatry.

[39] Chlebek J., Doskocil I., Hulcová D., Breiterová K., Šafratová M., Havelek R., Habartová K., Hošt’álková A., Volštátová T., Cahlíková L. (2016). Cytotoxicity of naturally occurring isoquinoline alkaloids of different structural types. Nat. Prod. Commun..

[40] Zhu J.P. (1998). Chinese Materia Medica: Chemistry, Pharmacology and Applications.

[41] Subaiea G.M., Aljofan M., Devadasu V.R., Alshammari T.M. (2017). Acute toxicity testing of newly discovered potential antihepatitis B virus agents of plant origin. Asian J. Pharm. Clin. Res..

[42] Lineweaver H., Burk D. (1934). The determination of enzyme dissociation constants. J. Am. Chem. Soc..

[43] Cheung J., Rudolph M.J., Burshteyn F., Cassidy M.S., Gary E.N., Love J., Franklin M.C., Height J.J. (2012). Structures of human acetylcholinesterase in complex with pharmacologically important ligands. J. Med. Chem..

[44] Forli S., Huey R., Pique M.E., Sanner M., Goodsell D.S., Olson A.J. (2016). Computational protein-ligand docking and virtual drug screening with the AutoDock suite. Nat. Protoc..

[45] Crivori P., Cruciani G., Carrupt P.A., Testa B. (2000). Predicting blood-brain barrier permeation from three-dimensional molecular structure. J. Med. Chem..

[46] Cahlíková L., Pérez D.I., Štěpánková Š., Chlebek J., Šafratová M., Hošt’álková A., Opletal L. (2015). In vitro inhibitory effects of 8-*O*-demethylmaritidine and undulatine on acetylcholinesterase and their predicted penetration across the blood–brain barrier. J. Nat. Prod..

[47] Muehlbacher M., Spitzer G.M., Liedl K.R., Kornhuber J. (2011). Qualitative prediction of blood–brain barrier permeability on a large and refined dataset. J. Comput. Aided Mol. Des..

[48] Abraham M.H., Takács-Novák K., Mitchell R.C. (1997). On the partition of ampholytes: Application to blood–brain distribution. J. Pharm. Sci..

[49] Carpenter T.S., Kirshner D.A., Lau E.Y., Wong S.E., Nilmeier J.P., Lightstone F.C. (2014). A method to predict blood-brain barrier permeability of drug-like compounds using molecular dynamics simulations. Biophys. J..

[50] Trott O., Olson A.J. (2010). AutoDock Vina: improving the speed and accuracy of docking with a new scoring function, efficient optimization, and multithreading. J. Comput. Chem..

[51] Liu B., Wang L., Jin Y.-H. (2007). An effective PSO-based memetic algorithm for flow shop scheduling. IEEE Trans. Syst. Man. Cybern. B Cybern..

