# Supplementary material for: In Vitro and In Silico Acetylcholinesterase Inhibitory Activity of Thalictricavine and Canadine and Their Predicted Penetration across the Blood-Brain Barrier
Source: Molecules. 2019 Apr 5;24(7):1340. doi: 10.3390/molecules24071340 (PMC6480038; doi:10.3390/molecules24071340)
Supplement: Supplementary file 1 [file molecules-24-01340-s001.pdf]

Supplementary Materials

In Vitro and *In Silico* Acetylcholinesterase Inhibitory Activity of Thalictricavine and Canadine and Their Predicted Penetration across the Blood-Brain Barrier

Jakub Chlebek [1,*], Jan Korábečný [2,3], Rafael Doležal [2,4], Šárka Štěpánková [5], Daniel I. Pérez [6], Anna Hošťálková [1], Lubomír Opletal [1], Lucie Cahlíková [1], Kateřina Macáková [1], Tomáš Kučera [3], Martina Hrabinová [2,3] and Daniel Jun [2,3]

1 ADINACO Research Group, Department of Pharmaceutical Botany, Faculty of Pharmacy, Charles University, Akademika Heyrovského 1203, 500 05 Hradec Králové, Czech Republic; HOSTA4AA@faf.cuni.cz (A.H.); opletal@faf.cuni.cz (L.O.); cahlikova@faf.cuni.cz (L.C.); macakovak@faf.cuni.cz (K.M.)

2 Biomedical Research Center, University Hospital Hradec Králové, Sokolská 581, 500 05 Hradec Králové, Czech Republic; korabecny.jan1@gmail.com (J.K.); rafael.dolezal@gmail.com (R.D.); martina.hrabinova@unob.cz (M.H.); daniel.jun@unob.cz (D.J.)

3 Department of Toxicology and Military Pharmacy, Faculty of Military Health Sciences, University of Defense, Třebešská 1575, 500 01 Hradec Králové, Czech Republic; tomas.kucera2@unob.cz (T.K.)

4 Center for Basic and Applied Research, Faculty of Informatics and Management, University of Hradec Králové, Rokitanského 62, 50003 Hradec Králové, Czech Republic

5 Department of Biological and Biochemical Sciences, Faculty of Chemical Technology, University of Pardubice, Studentská 95, 532 10 Pardubice, Czech Republic; Sarka.Stepankova@upce.cz (Š.Š.)

6 Centro de Investigaciones Biológicas, Avenida Ramiro de Maetzu 9, 280 40 Madrid, Spain; dperez@cib.csic.es (D.I.P.)

* Correspondence: jakub.chlebek@faf.cuni.cz; Tel.: +420-495067232; Fax: +420-495067162 (J.C.)

Table S1. Permeability in the PAMPA-BBB assay for nine commercial drugs (used in the experiment validation), compounds **1** and **2** and their predictive penetration into the CNS [1].

compound	PAMPA-BBB (*Pe*; 10^{-6} cm.s^{-1})		prediction
	reported permeability [2]	experimental permeability [3]	
atenolol	0.8	0.1±0.1	
caffeine	1.3	0.3±0.1	
desipramine	12.0	12.7±0.4	
enoxacin	0.9	0.2±0.1	
hydrocortisone	1.9	0.7±0.6	
piroxicam	2.5	0.2±0.2	
promazine	8.8	13.3±0.4	
testosterone	17.0	22.5±2.5	
verapamil	16.0	18.5±1.2	
**1**		2.5 ± 0.1	CNS ±
**2**		5.0 ± 0.3	CNS +

[1] PBS/EtOH (70:30) was used as a solvent. [2] Reference (Di et al., 2003). [3] Data are the mean ± SD of three independent experiments.
